# Utilising Emotion Monitoring for Developing Music Interventions for People with Dementia: A State-of-the-Art Review

**DOI:** 10.3390/s23135834

**Published:** 2023-06-22

**Authors:** Jessica G. J. Vuijk, Jeroen Klein Brinke, Nikita Sharma

**Affiliations:** Pervasive Systems, Faculty of Electrical Engineering, Mathematics and Computer Science (EEMCS), University of Twente, 7522 NB Enschede, The Netherlandsn.sharma@utwente.nl (N.S.)

**Keywords:** pervasive sensing, device-free sensing, emotion detection, dementia, music

## Abstract

The demand for smart solutions to support people with dementia (PwD) is increasing. These solutions are expected to assist PwD with their emotional, physical, and social well-being. At the moment, state-of-the-art works allow for the monitoring of physical well-being; however, not much attention is delineated for monitoring the emotional and social well-being of PwD. Research on emotion monitoring can be combined with research on the effects of music on PwD given its promising effects. More specifically, knowledge of the emotional state allows for music intervention to alleviate negative emotions by eliciting positive emotions in PwD. In this direction, the paper conducts a state-of-the-art review on two aspects: (i) the effect of music on PwD and (ii) both wearable and non-wearable sensing systems for emotional state monitoring. After outlining the application of musical interventions for PwD, including emotion monitoring sensors and algorithms, multiple challenges are identified. The main findings include a need for rigorous research approaches for the development of adaptable solutions that can tackle dynamic changes caused by the diminishing cognitive abilities of PwD with a focus on privacy and adoption aspects. By addressing these requirements, advancements can be made in harnessing music and emotion monitoring for PwD, thereby facilitating the creation of more resilient and scalable solutions to aid caregivers and PwD.

## 1. Introduction

At present, over 50 million individuals worldwide are experiencing the direct effects of dementia [1]. Dementia is a neurodegenerative disorder which not only impacts cognitive ability, but also interferes with Activities of Daily Living (ADL) and the emotional stability of people with dementia (PwD). As a person gets older, their risk of developing dementia increases [2]. With the progression of dementia stages, PwD may become increasingly dependent on informal caregivers (friends, family, or relatives). Providing informal care has detrimental effects on the physical health, emotional well-being, and financial stability of informal caregivers. This is particularly true when providing caregiving for older adults with dementia, because as their physical abilities reduce due to age, they also become dependent on formal and informal caregivers for cognitive functioning such as memory (forgetting to take medicine), awareness (lack of awareness of past, present, and future events), judgment (indecisiveness), and more. Usually, these cognitive impairments may be accompanied and preceded by changes in mood or behavior, thus impacting emotional stability [3].

Current estimates are that the number of PwD is expected to rise to 78 million by 2030 [4] due to the growing older adult population and the increasing average lifespan. This increment in older adults with dementia demands an increase in the availability of the aforementioned (in)formal care. The focus of this review is on sensor-based systems that assist in delivering emotional care for PwD to mitigate the effects of dementia on emotions. Previous studies report that PwD may have reduced control over their emotions depending on the type and level of dementia [5]. PwD may display a wide range of emotions, ranging from negative to positive. Negative emotions may cause mental discomfort, which potentially negatively affects their surroundings. On the other hand, positive emotions may enhance social interactions and might enhance cognitive or physical abilities [3].

At the moment, no cure exists for dementia, and while pharmaceutical approaches exist to mitigate progressive dementia symptoms, they may come with adverse side effects. Therefore, non-pharmaceutical alternative interventions are desirable. According to the literature, music could help address the limited control of emotions in PwD. Music is found suitable as PwD can learn, recognise, and respond to music, despite their cognitive decline [6,7,8,9,10,11,12]. Additionally, music allows for communication without using proper language/verbal communication [6,13], which is a reoccurring challenge in the progression of dementia. The effects of music mentioned in the literature include, amongst others, alleviation of emotional and behavioral disturbances [14].

Currently, the costs for care are estimated to be around 1% of the gross domestic product [15]. In order to limit the effects of dementia on society, there should be a focus on solutions that minimise the cost for training of caregivers. To address these implementation challenges, low-cost technologies that require little to no effort and understanding from caregivers can be used to assist in recognising the emotions of PwD. Potential solutions could be found in combining music and emotion monitoring in smart systems to monitor the subject’s emotional state continuously to act upon changes promptly and accordingly using fitting musical items. These tools can potentially relieve caregivers from constantly checking PwD at regular intervals. Thus, such systems could address different emotional states appropriately in a time- and cost-efficient manner, reducing the workload of (in)formal caregivers.

Emotions can be recognised by monitoring psychophysiological markers (e.g., pulse, sweat conductance, and breathing) and facial expressions [16]. Various state-of-the-art sensing and monitoring systems exist. These include camera-based solutions or (a combination of) wearables such as smart watches, jewellery, or smart clothing. Although there is a shift towards wearables these days, this work instead focuses on positioning sensors in the environment, rather than on the body. This is deemed appropriate given the potential limited mobility of older PwD and it eliminates the need for the person with dementia or caregiver to wear the sensors, which can be inconvenient or forgotten.

Ideally, a low-cost sensing system combining emotion monitoring and music will be used in the future to address the pressure of dementia on society. However, to the authors’ knowledge, no such system is available yet. A first step in this direction is gaining insight into emotion monitoring systems and the role of music in the emotion management of PwD. Therefore, this paper conducts a state-of-the-art review of these topics.

The remainder of this work is organised as follows: Section 2 describes how the literature has been collected systematically in order to address both sensing systems for emotional state monitoring as well as the effect of music on people with dementia. Thereafter, Section 3 reports on the found literature in order to obtain an overview of existing knowledge on the topics and address ethical challenges. Section 4 concatenates the information that was found and critically reflects on it, unveiling the challenges in the topic. Finally, Section 5 ends this work with a conclusion in the shape of recommendations to improve the quality of future research as well as a recommendation for a research topic opportunity.

## 2. Methodology

This section describes the process of identifying the relevant literature. The approach is based on the Preferred Reporting Items for Systematic Reviews and Meta-Analyses (PRISMA) [17]. After duplicate removal from different databases, the titles and abstracts were screened. Finally, the resulting set of literature was read entirely and assessed on eligibility.

**Figure 1:**
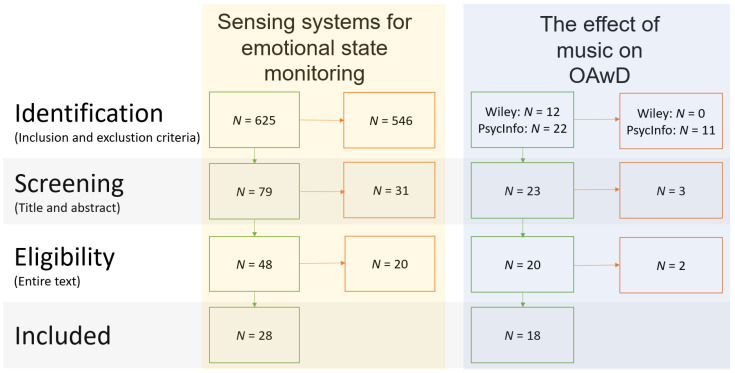
Article selection overview.

This state-of-the-art review consists of research into two domains: social sciences and exact sciences. Therefore, a collection of databases has been consulted: Scopus, PsycInfo, and Wiley. The aforementioned process was applied to both domains in order to identify the most relevant papers to this study. This process is depicted in Figure 1 and described in more detail in Appendix A.

### Data Extraction

Key elements have been identified to help guide the data extraction in order to determine the state-of-the-art methods, current limitations, and open challenges. For sensing systems enabling emotional state monitoring, the key elements for data extraction include the activity or emotion being monitored, the sensors and algorithms deployed, the study environment, the participant(s) and their demographics, and the main findings. For the effect of music on PwD, this includes the sample size and their demographics (age, gender, and dementia severity), methodology aspects (e.g., individual or group approach, active or passive music interactions, and the type of music), and lastly, the main result(s) of the study. The results can be found in Appendix B and Appendix C.

## 3. Results

### 3.1. Sensing Technologies for Emotion Recognition

In 2010, Taleb et al. [18] mentioned that the merging of pervasive healthcare and affective computing was a new phenomenon. This implies that the use of pervasive systems, in which smart technology is ubiquitous and seamlessly integrated into daily lives [19], in healthcare for affective applications started around 2010. In the following years, many different ways to measure their effects have been studied. Esch et al. [16] mentioned three channels: speech (1), face and body gestures (2), and internal physiological changes (3). However, recent studies have discussed additional methods. The reported tools are listed below, grouped into three categories: self-reporting, bodily sensors, and device-free sensing. Table 1 provides a schematic overview of which (combined) methods were used in the reviewed papers in this work. Additionally, Table 2 groups these reviewed papers based on their use of machine learning, or more specifically deep learning, to process the data obtained using the sensors. Deep learning is a subset within machine learning that trains (layered) neural networks to recognise patterns in large sets of data, while machine learning encompasses a broader range of algorithms and techniques to perform tasks based on large and problem-specific data [20]. More details on the algorithms used can be found in Appendix C. Systems commonly combine multiple measuring techniques to improve the performance of the classification. Although multiple studies include self-reporting, it should be noted this is mostly done for labeling and validation purposes of the models, rather than being used for the purpose of emotion detection itself.

Zangerle et al. [50] make a critical note that many studies sensing emotions take place in laboratory settings. This is also reflected in this work, as can be seen in Table 3. Therefore, it is unclear how these findings translate to actual real-life settings.

#### 3.1.1. Self-Reporting for Emotion Recognition

Self-reporting is a well-known tool to measure a subjective construct such as emotion; subjects are asked to fill in questionnaires on how something affects them. Examples of validated questionnaires include State-Train Anger Expression Inventory 2 (STATE 2) and UWIST Mood Adjective Checklist (UMACL) as, for example, used by Fairclough et al. [33]. Reports can be voice recorded or written (both digital and paper), depending on the study design. However, subjects may decide not to share accurate feelings or thoughts on some topics. Another concern is that subjects may not complete the survey at the intended moment, or omit it completely [49]. Moreover, a subject might unintentionally recall the feelings or experiences of a past event differently later. These biases can influence the objectivity and validity of the results. Yet, the benefit of self-reporting is the ease of implementation [49]. Work by Thorpe et al. [32] illustrates the feasibility of implementation of self-reporting as a measurement tool in a system for PwD.

#### 3.1.2. On-Body Sensors for Emotion Recognition

Research has shown a strong relation between affective states and physiological reactions [18,49,51]. These physiological changes can be measured using biosensors. The biosensors and measured parameters found during this systematic review were electroencephalography (EEG), electromyography (EMG), electrodermal activity/response (EDA/EDR), heart rate and its variability, blood pressure, weight, oxygen saturation, skin conductance, temperature, body composition measures, respiration rhythms, motion, and blood glucose. However, it should be noted that there are more biosensors and parameters.

These sensors are frequently implemented in clothing, accessories, or on the body. Such sensor networks are called body area networks (BANs) [16] or body sensor networks (BSNs) [51]. This allows for continuous measuring and real-time decision making. Since wearable sensors often have low power consumption and research in portable batteries has enabled longer battery lives, they offer efficient and cost-effective solutions, while outside the scope of this review, it should be noted BANs offer the opportunity to connect individual BANs in order to monitor groups.

Another on-body sensor is the individual’s (smart)phone, which comes with many benefits. As they are well-integrated into society, they could be counted as an unobtrusive sensor to measure various parameters. Opportunities presented by data extraction from (smart)phones include two types of information: behavioural and physical. Behaviour information includes, amongst others, the frequency of calling or texting, screen time (general or application specific), tracking user behaviour (online and offline), and the language used in texts or posts. On the other hand, physical information includes, but is not limited to, acceleration, velocity, rotation, and light intensity. Aside from the diversity of data collected from phones, the acceptance of subjects may also be higher, due to the possibility of penalisation and lack of any stigma when compared to other assistive technologies [32].

#### 3.1.3. Device-Free Sensing for Emotion Recognition

A device-free solution for measuring psychophysiological changes involves the creation of a smart environment that utilises sensors embedded in everyday objects surrounding a subject (e.g., furniture or doorsteps). These sensors enable the environment to track and monitor the behaviour and habits of the user in an non-intrusive, adaptable, and dynamic manner. These systems are called dense or mesh sensor networks [16]. These networked sensor systems consist of two elements: transducers and tranceivers. The former are used for data collection (sensors), while the latter are used to communicate the collected data [16]. By analyzing the data collected by the sensors, the system can determine changes in the subject’s physiological state, movements, and habits. The benefits of these sensor networks include self-organisation, scalability, reliability, power efficiency, and adoption in complex and dynamic environments.

A specific sensor often used in such a network is a camera. These cameras are often combined with algorithms to classify a user’s emotion based on facial expressions. The stimulus can be a picture or a video [49]. Although both pictures and videos can be used to recognise emotions directly using machine learning, videos also allow for recognition of certain gestures or movements, such as nodding or head shaking. However, emotion classifications based on facial analysis might not always be accurate as people can display expressions that are not in line with their emotions [49].

Another popular sensor that could be added to the environment is a microphone, as human speech contains elements that express emotions. There are several ways to extract emotion from speech. First of all, the content of speech could be analysed [52]. Negative terms could indicate negative feelings, while positive topics could be linked to positive emotions. However, language is one of the components that could be affected by dementia. Another downfall of focusing on content is the lack of universality [49], as systems are only applicable to contexts where the same language is used that the system has been trained on. Nonetheless, it is a low-cost and non-intrusive implementation [49]. To negate these limitations of speech, other elements of the voice could be used to estimate the affective state of the subject. Opportunities include the analysis of pitch, prosody, and other acoustic elements [52]. Additionally, sounds expressing emotions (e.g., crying and laughter) are universal and can therefore be recognised regardless of the language on which a model is trained.

Similar to (smart)phone interactions, it is also possible to track a subject’s computer usage. Examples include the search terms applied in a browser, the time spent on certain web pages, or the used applications. Similar to smartphones, computers also allow for the subject to engage in social media, something that more than half of the world engages in [53].

In addition to using a single computer as a measurement technique, it is also possible to analyse network traffic. Mobile network data have been studied regarding their ability to infer affective states. Although this research is still in early stages, preliminary results show that it is possible to infer the social mood of a collection of people in a city using this type of data [49].

In addition to the aforementioned audiovisual techniques, infrared sensors are being researched for monitoring PwD. Infrared sensors are devices that detect and measure infrared radiation. They are designed to perceive and capture the heat energy emitted by individuals in their vicinity. By detecting and converting infrared radiation into electrical signals, these sensors can be used to identify movement or patterns of the PwD. These can be larger movements such as walking or smaller movements such as respiration or heart rate [54].

The previously mentioned options are encountered regularly in the literature. However, upcoming alternatives should also be explored further when considering music systems to address emotion regulation, such as measuring whether older adults move along to the beat of music. Another idea is to track their sitting style [55]. Raja and Sigg [56] explored the opportunity of using radio waves to detect emotions. The findings suggested it is a potential technique to track bodily movements and posture device free. This means that it would be suitable for measuring body movements (moving to the beat) and posture (slouching). Another suggestion is tracking the eyes to measure attention and workload [34]. Strong attention or a high workload could mean an individual is not able to relax. This assumption would need to be supported in the literature or by an experiment.

### 3.2. Data Processing Methods for Emotion Recognition

Previously, it has been described how sensors can be used to obtain information about the user or its context (Section 3.3.3). However, raw data are often not suitable for data analysis. Raw data are regularly affected by differences in sampling frequencies, signal noise, and other disturbances [35]. Preprocessing techniques are usually applied to make the data more suitable for analysis, and the used technique varies per sensor input and desired output. A typical process for emotion recognition [49] is to extract features from the collected data, which are then used in a classification algorithm to classify or predict the emotional state.

Knappmeyer [57] provides a general model of a context-aware system cycle. The cycle consists of four stages. In the first stage, data are collected from sensors for context acquisition. In the second stage, the model that has been generated is analyzed to identify which pieces of the information are most relevant. In the third stage, algorithms are used to reason, make decisions, or form recommendations. In the fourth and last stage, the context-aware system uses the context information to adapt its behavior. In this review, attention is directed towards a context-aware system targeting the mental context, and thus the information about the subject’s internal state. Such a system would go through a continuous loop where data on indicators of the emotional state are collected, analysed, and aggregated to decide which musical item to play and learn from the impact this item has made.

Deep learning or machine learning algorithms are often applied to map raw sensor values (often numerical values corresponding to a voltage output) to emotion classifications or estimations. To illustrate the huge variation in approaches of applications, three main categories were considered:

- Statistical: ANOVA [35,37,41], multi-factorial analysis [40], principle component analysis [34,41], and (Spearman) correlations [42].
- Classical machine learning: Bayesian or simple clustering [49], decision trees [33,36,37], extreme gradient boosting [36], Fisher linear projection [18], hidden Markov model [18], kNN [33,41], linear or quadratic discriminant [33,41], MADM algorithm using Bayes rule [18], and SVM [18,36].
- Deep learning: CNN [39], genetic programming [45], MLP [41], probablistic neural networks [41], and RNN with LSTM [38].

### 3.3. Music and Dementia

The available knowledge in the area of music and dementia is vast. Therefore, to provide a comprehensive and structural understanding, this section first explains the association of music and the brain to provide explanations on why or how the brain responds to music. Following that, state-of-the-art research on music as an intervention for therapies for PwD is highlighted. Lastly, models of affect that describe emotions are discussed.

#### 3.3.1. Music and the Brain

Music memory is a part of the brain that seems to remain (partially) intact in individuals diagnosed with dementia [12,14,58,59]. Jacobsen et al. [60] explain the preservation of music memory. Nonetheless, disease-associated alterations in the processing of music and emotions may be observed [61,62].

Music stimulates the activation of multiple areas within the cerebral cortex, which might be linked to the changes seen in emotion and behaviour in PwD [63]. Emotion in music is known to be a complex system distributed throughout the brain [14,61]. While the exact mechanisms involved in the emotional processing of music are not fully understood yet [59,62], it is known that such mechanisms used to analyse music are intimately linked to pleasure and reward systems [64,65]. This could explain why music has the aforementioned positive effects on PwD. Other explanations for the positive effect of music on PwD include the emotional and evocative elements music has to elicit repressed feelings [61,63]. Lin et al. [63] mention the effect pitch has on the autonomic nervous system (ANS), where a low pitch stimulates a relaxing result. Additionally, familiar songs may help PwD access memories otherwise not accessible any more [66].

#### 3.3.2. Music Therapy

Music therapy is defined as “the use of music and/or of its components (sound, rhythm, melody, and harmony) by a qualified music therapist, in individual or group relationships, in the context of a formally defined process, with the aim of facilitating and promoting communication, relationships, learning, mobilisation, expression, organisation and other relevant therapeutic goals intended to meet physical, emotional, mental, social and cognitive needs” (p. 294) [13]. Music interventions can help reduce isolation, and in the context of dementia, it can alleviate secondary symptoms (e.g., personality changes and inappropriate social behaviour) [67]. Note that the term “music therapy” is often applied incorrectly in research [13]; not all studies make use of a qualified music therapist, which is one of the requirements for music therapy. Hence, some studies reviewed in this work may use this term, while it may not be appropriate. However, these works were still included, as non-formal music interventions are of general interest. Therefore, no reflective comments will be made in regards of this aspect.

A distinction is made between passive/receptive and active music interventions. Active music interventions require active involvement of the subject, such as clapping or playing an instrument [68]. On the other hand, receptive music interventions do not require active participation. This includes listening to music and expressing the effects on the emotional state. Each serves its own purpose: active music interventions promote general and social interactions, while passive music interventions lessen agitation [63].

Reasons to consider music interventions over pharmacological treatments include the limited effectiveness of medication [6] and the avoidance of side effects [6,63]. Music interventions are in particular useful for PwD as they are often able to learn, recognise, and respond to music, even when dementia has progressed into its later stages [6,7,8,9,10,11,12]. Additionally, music allows communication without using proper conversational language [6,13], which can be a recurring challenge in the progression of dementia. Thus, music interventions could address the double dissociation between language and music as described by Polk and Kertesz [69]. Double dissociations can be described as two mental processes that are related to each other, but have been shown (often due to brain damage) to function independently from each other.

Dorris et al. [70] put together a systematic overview on the effect of active music interventions on PwD with mild or moderate severity. The overall finding supports a significant effect on the cognitive function of PwD or subjects with mild cognitive impairment. These findings are found in a wide range of studies varying heavily in methodology; some have interventions taking 30 min to two hours, being performed one to five times a week in groups or individually and lasting from four to forty weeks, as well as the variation in tools used for measuring outcomes. In addition, individual studies have shown positive effects on the quality of life and the mood of PwD [66,71,72]. These results are in line with a meta-analysis performed in 2013 [68]. However, the earlier results showed larger effects and made a distinction between affective, behavioural, cognitive, and physiological outcome measures. For behavioural, cognitive, and physiological outcomes, they found large positive effects (above 0.6), while for the affective measures, they found medium effects (between 0.2 and 0.6). The increased effect could be explained by differences in the methodology and quality of the conducted experiments.

Besides the results summarised by Dorris et al. [70], the literature has also shown that music interventions can:

- Reduce agitated behaviour [63,73,74,75,76];
- Reduce behavioural disorders [6,13,66];
- Enhance emotional relaxation [6,72,73,77];
- Increase positive behaviours (laughter and rhythmic movements) [73,75,77,78,79];
- Stimulate autobiographical memories [13,80];
- Target depression symptoms [66,81];
- Create inter-personal interactions [13,63,66,71,72].

In contrast to the previously mentioned results, Solé et al. [82] found a decrease in social interaction and a (non-clinically significant) decrease in the quality of life. Still, an increase was found in the subscales regarding emotional well-being and personal development. The interpersonal relations decreased. However, due to the small sample size of this study (*n* = 16), more research is required to verify the conclusions.

An additional benefit of music interventions that has not been addressed yet is the positive impact on caregivers. Research has shown that it can both reduce caregivers’ distress [6] and improve their well-being [83,84]. Furthermore, Baird and Thompson highlight the added benefit of music on caregivers [12], which includes both formal and informal caregivers. Positive effects such as mood or behavioural changes, feelings of inclusion or support, and improved social connectedness are observed in the caregivers. Music does not only have a positive effect on the PwD and caregiver individually, but also positively affects their relationship [72].

#### 3.3.3. Emotional Models

Music is able to affect people with dementia, as outlined in Section 3.3, including moods, emotions, and memory. Attention is given to short-term effects, as popular songs are often only about three minutes long. Therefore, emotion is one of the suitable indicators of the effect of music.

In order to study the effects on emotions, it is important to consider models of emotions. A commonly known model is a categorical model, which assigns every emotion to its own distinct category. A widely recognised categorical model is defined by Ekman et al. [85], which distinguishes six basic emotions: anger, disgust, fear, joy, sadness, and surprise. Although these emotions are universal, cultural differences could influence when, to whom, and how they are expressed, as well as the ways in which they are discussed and described.

Another categorical model is Plutchik’s wheel of emotions [86], which differentiates between eight basic emotions: anger, anticipation, joy, trust, fear, surprise, sadness, and disgust. The wheel is comparable to the colour wheel: it consists of primary emotions, the ones that were just mentioned, but also secondary and beyond. Emotions closer to the centre are more intense, and moving away from the centre reduces intensity. Moreover, (polar) opposite emotions are placed opposite of one another in the circle.

In contrast to this categorical (discrete) model are continuous approaches, where emotions are put on a multi-dimensional coordinate system. The circumplex model by Russell et al. [87] is a prominent example of this. In this case, emotions are positioned on a coordinate system with two continuous axes (arousal and valence). By describing emotions this way, the authors contrast the basic models that build on the theory that each emotion is subserved by a discrete and independent neural system [88]. Differences between various models and an in-depth analysis of emotion representation are described by Peter et al. [89].

### 3.4. Ethical Considerations

With the growing interest in monitoring systems, both in academia and industry, it becomes increasingly more important to assess the ethics and consider privacy preserving techniques. Sensors are able to collect sensitive data (e.g., images, GPS coordinates, or vital signs of a subject [90]) invisibly and continuously. Wu et al. [35] mentions that one should carefully think about privacy issues from the start of the design process to produce good and safe technology. Similarly, Kanjo et al. [49] stress the risks of privacy invasion when collecting varied and extensive amounts of information. Especially in the context of cloud computing, they recommend to take extra protection measures in order to preserve data integrity. Moreover, they stress it is important to guarantee the user’s privacy when recruiting participants for experiments. One of the methods by which attackers gain sensitive data is by training classifiers to predict private data belonging to a known community [90].

Different sensors and communication strategies come with different privacy concerns. Individuals are easily recognised through audiovisual sensors by other humans. Additionally, their behaviour and relationships (e.g., private conversations or living habits) are more easily inferred. On the other hand, sensors that measure physiological events (e.g., heart rate, brain activity, and skin conductance) gather data that are harder to link directly to an individual, but may contain more privacy sensitive information that should not be shared. For instance, a person may feel uncomfortable knowing that an affective state (e.g., ashamed or nervous) is shared with a relative, especially in cultures that try to minimise showing emotions. This closely links to a strong bias within self-reporting, where people sometimes decide to not share their actual emotions.

Over time, multiple approaches that prevent others from mining critical information have been developed [16]. Saxena et al. [91] proposed a secure and privacy-preserving data aggregation scheme using encryption and a cryptosystem. Several other data privacy techniques are addressed by Baccour et al. [90]. Moreover, Esch recommends to use a privacy-by-design approach, meaning the privacy requirements are collected first, after which the design should adhere to these. Langheinrich [92] also identified six notions to be taken into account for privacy preservation in ubiquitous systems: (1) notice, (2) choice and consent, (3) anonymity and pseudonymity, (4) proximity and locality, (5) adequate security, and (6) access and resource. McNeill [93] adds to this with seven key concepts to address the privacy perceptions of older adults: (1) self-protection, (2) autonomy, (3) emotional release, (4) confiding, (5) social identity, (6) self-concept, and (7) protecting others.

Anonymity is another element that could address privacy concerns in a pervasive system. Chakravorty et al. [94] proposed a method that transforms personal data into hash values before the data are used in any analysis. This means it is harder to trace data back to an individual during analysis. However, it should be noted that hashing the same number results in the same hash value, which could still be considered a unique identifier from which data could be inferred.

Moreover, one should be careful to not cause a “big brother” effect [95], wherein people feel uncomfortable due to a perceived sense of constantly being watched. Not only is this a negative experience for the user, but this could also cause a Hawthorne effect, meaning people may change their behaviour due to this perceived sense of being watched [96].

Another ethical concern to take into consideration is the effect that pervasive systems can have on a (group of) person(s) [16,49]. Negative effects, such as over-dependence on the system, decreased trust, or minimised interaction with others, should be prevented.

## 4. Discussion

In this section, we further elaborate and discuss the observations made in the results and challenges for both emotion detection effect of music on people with dementia (Section 3). The main challenges are summarised in Table 4.

### 4.1. Emotion Detection

Given the short duration of much popular music, studying the effect of music by analysing affects or emotions is an appropriate method. Different models of affect exist, including both discrete categories and continuous coordinate systems. The majority of studies have a preference for distinct categories, as can be seen in Appendix C and Taleb et al. [18], as these categories can form the output of an emotion detection system.

Based on the results of this review, it is implied that there is no universal solution. This results in the literature often targeting a niche application, limiting the possibilities to generalise the promising results to PwD or beyond [18]. Therefore, it is important to follow human-centered design approaches to enable customisable solutions to adhere to the actual needs and pathologies of PwD [97,98]. In order to achieve generalisability, it is important to consider what data are available or required and which model(s) fits the input data. Moreover, the goal of the system may also impact the choice of solution, as this may vary between binary (stress versus distress), categorical (classifying basic emotions), or continuous (estimations of valence and arousal levels) outputs. Zhang et al. [99] provides an extensive tutorial and review on emotion recognition using multimodal data. The authors also highlight commonly used datasets, preprocessing techniques, and machine learning algorithms. Additionally, the authors provide a list of appropriate and frequently used evaluation metrics used in AI: precision, sensitivity (also known as recall), specificity, and F-score.

Similarly, ground truth data or general data formatting is not standardised. Kanjo et al. [49] mention the limited availability and generalisability of datasets. Moreover, they suggest to create spatiotemporal visualisations in order to generalise system evaluations and dissemination of information.

Another challenge is to move from a laboratory setting to real-life in-home situations, as currently the majority of studies take place in the lab. Kanjo suggests to perform longitudinal, in-field experiments while using mixed methods inspired by the social sciences in order to gain a better understanding of such systems [49]. However, this also comes with additional challenges, such as the limited control over environmental or other external factors by the researcher, such as social interaction [34]. This could partly be dealt with when middleware (software connecting an operating system and its applications) proposals support context modelling and other forms of context awareness (e.g., psycho-emotional and group awareness) [18].

The input is created by sensors, can vary widely, and each type has its own contributions and drawback. Most studies decide to combine different types of measuring to increase accuracy (Table 1). Self-reporting is a technique that may not work with PwD, as dementia may cause cognitive limitations that prevent the subject from answering the prompt. It could be perceived as subjective and obtrusive, as it requires active user input and sharing personal thoughts and experiences. The recommendation is to use it solely for training and validation purposes. Additionally, the aim is to lighten the burden on informal and formal caregivers, so it is also undesirable to forward the responsibility of answering these questionnaires to them. However, it is a useful tool to obtain a ground truth for emotion recognition [95]. For this reason, self-reporting is commonly used, despite its drawbacks.

From Table 1, it may be inferred that on-body sensors are the most used sensor type. Frequently used sensors include ECG, EEG, and EDA. This type of measuring comes with many benefits, as on-body sensors are objective, continuous, and mobile. However, on-body sensors may be perceived as obtrusive, visible, and uncomfortable. Although there are a wide-range of solutions to measure psychophysiology, BAN should be used in limited settings, given the aforementioned drawbacks. Mahmud [36] adds a concern regarding noninvasive monitoring of physical biomarkers. While these are often integrated into wrist-worn devices, they are susceptible to motion artifacts, resulting in noisy data. This implies these may not be the most optimal sensors. The concerns are generalised beyond the effects of motion, namely to costs, data accuracy, mobility, fashion, and reliability. Research by Betella et al. [34] adds to this, stating that wireless sensors may have other challenges, namely stable wireless connectivity or sensor sizing. In short, while these sensors are sensitive to noise, they offer access to psychophysiologal data otherwise unavailable, and their success in providing data for emotion detection can be seen in the literature.

Methods that circumvent the physical obtrusiveness of on-body sensors are (smart)phones, computers, and social media usage. Given their omnipresent use in modern life, they are a suitable solution in many applications. This information can be analysed both per individual and as a group or community [49]. It should be noted that this solution may not be suitable for real-time classifications, as users may decide to post pictures of an event belatedly. Therefore, conclusions should be drawn with caution. At first sight, another concern might be the relevance of such methods for PwD, as dementia is most common amongst older adults, and the use of smartphones and computers amongst older adults is often expected to be limited. However, statistics have shown that around 52–86% of older adults use (smart)phones in the Netherlands in 2019, 81% use a computer, and 40–76% are on social media [100]. Additionally, people familiar with technology will soon enter the range of age of older adults. Therefore, the use of smartphones, computers, or social media as classifier inputs should not be discarded based on the concern that older adults may not use these devices and services.

Moreover, smartphones and other wearable technologies have shown promising results when monitoring PwD. It is even mentioned that PwD use such devices as reminder or notification tools. However, the experiments conducted by Thorpe et al. [32] contain a limited sample size and only include people with early-stage dementia. Hence, the generalisation to the larger population of PwD and dementia stages should still be studied more. The validity of these insights should definitely be explored for people with late-stage dementia, as these may be the wrong target audience for using smartphones and computers as measurement tools.

Audiovisual technologies have shown promising results in device-free emotion recognition. Both speech and facial analyses are highly effective techniques to obtain accurate results on emotion estimation. However, they come with great concerns regarding privacy. When using a camera, a person and their activities are easily recognised by other humans. For microphones, there are different concerns: a person may be identified indirectly by their voice and private conversations could be recorded. This information could be intercepted by malicious third parties, which may lead to dire consequences. Additionally, the perception of being recorded may result in a negative experience or in a change in behaviour. Therefore, their application in healthcare should be minimised. Besides, expressions (both facial and linguistic) change in PwD, which limits the applicability of the aforementioned techniques. Recordings could still be used by specialists to monitor changes over longer periods of time, if the proper ethical consent is obtained. Options that remain include the use of a microphone to detect expressions of human emotions (e.g., crying and laughter) or environmental noises by filtering out the human speech frequency.

In conclusion, it is strongly discouraged to apply sensing techniques that are obtrusive (both physically and ethically) or that require an active input from the user. Instead, it is advised to apply sensors to the environment and monitor PwD indirectly. An example of such an implemention is infrared sensors, as they can gather data pervasively and continuously. This is particularly suitable for PwD because of the large amount of time they spend at home.

The analytical method that should be applied in an emotion-aware pervasive system depends on the available data and the required output (discrete or continuous output). Moreover, system requirements (e.g., power consumption, resource constraints, and computational complexity) may determine the suitable method.

Different methods have been studied in the existing body of literature. However, it should be noted that most reported performances are gained from experiments in a laboratory setting. Performance could be affected when conducting the same experiments in real-life settings. Future research should move towards in-field experiments to validate the results found in the laboratory setting.

### 4.2. Music and Dementia

Various music intervention designs may be applied, as described in Section 3.3. However, the term “music therapy” should be used carefully, as it implies the intervention design has met certain requirements. Nonetheless, music interventions enable great opportunities to contribute positively to the quality of life for PwD. Both passive and active music interventions have shown a positive and strong effect on both PwD and (in)formal caregivers. The side effects of pharmacological treatments can be avoided when using music to treat behavioural and psychological symptoms of dementia (BPSD).

Music interventions vary strongly in design, as can be inferred from Appendix B. This results in a lack of standardisation or clear requirements in the field. Suggestions from Wac et al. [95] include a clearer direction regarding the number of participants. In order to address the challenge in advocacy and civic engagement, Kanjo et al. [49] suggests that users should be offered an incentive. This could help promote participation in research that collects (sensitive) data about them. Another challenge is the limited diversity in studies, which may be attributed to anonymity. Authors think about diversity in a variety of domains such as cultural, physical, educational, and other interpersonal differences. An illustration of this point is that most research regarding recommender systems is applied to Western music, which is why Rashmi and Kalpana [101] targeted Indian music in their study. Lin et al. [63] also highlight how most studies have taken place in Western countries and only few took place in other cultures. However, cultural differences regarding emotion expression or processing could bring different results to light. Therefore, future research should set frameworks for stricter requirements, improved reporting, and increased standardisation. However, given the promising results in existing studies, the authors recommend continuing research in this domain with the adjusted strategy, as this allows for implications and conclusions with a higher degree of confidence.

Another difficulty is the personal taste and preference of PwD, as this increases the complexity of effective design. Lin et al. [63] mention that the general age and background should be taken into account when deciding which music to use. Vasionyté and Madison [68] add to this by distinguishing more elements, such as the difference between live and recorded music. Another factor to consider is the (social) distance between the individual responsible for music selection and PwD, as well as the availability of personalised music choices. Lastly, music genre (e.g., classic, pop, or rock) and individual or group-based intervention may affect the effectiveness.

As a result of the heavily varying methodologies, multiple studies discuss the lack of methodological rigour or proper reporting in research on the effects of music on PwD [6,14,68,70,77,102,103,104,105]. Criticism includes the lack of precision or clarity in reporting, replicating the same experiments, or interpretation and comparison of different results. Guidelines for classification are not always used, meaning studies contain biases (e.g., small sample sizes, no randomisation, no control group, or the general lack of a blind assessor). In some research, the data used are incomplete.

It is important to consider the trade-offs in a system applying music intervention to improve emotional well-being in order to obtain the best results in such a system. An example of this is live music performing better than pre-recorded music, but at the same time being more expensive and only available to a limited audience. The compromise for PwD in this case would be to play recordings of live performances. However, from the studied works, it cannot be inferred whether recordings of live music obtain better results than studio sessions. Another trade-off is cost versus efficiency; given the expectation that the number of PwD will rise to 78 million by 2030 [4], it is very important that any proposed solution is cost-effective. However, if a solution is designed poorly or inefficiently, maintenance and repair may result in a higher cost over long-term deployment or intervention. Other considerations when designing music solutions are the choice of music intervention (active or passive) and the number of participants (individual or group). Group solutions may be more cost-effective compared to individual interventions, but may be less effective than individual interventions. Similarly, passive interventions may be more cost-effective due to automation, but active interventions may have their own positive effect on PwD (see Section 3.3).

Current research focuses mostly on the short-term effects of the proposed solutions and there is minimal research on the effects of long-term solutions [6]. The limited research that has considered long-term effects shows promising results, meaning it is worth spending resources towards this effort. Särkämö et al. [83] observed long-term cognitive, behavioural, and social benefits in PwD when formal caregivers implemented both active (singing) and passive (listening) musical activities. Besides the duration of the solution, the stage of dementia should also affect the scheduling of interventions for PwD, as it is preferrable to start therapy sessions in the early stages to obtain the desired effects [67]. If this is not feasible, more intense therapy sessions are suggested, which could be four to five short sessions a week instead of one longer one.

While music has shown positive effects on PwD specifically, it may also be suitable for emotion regulation in the general population. This means that a solution using music may be generalised to a larger audience. However, there are extra elements to consider when working with PwD when applying therapeutic elements and performing experiments. Halpern et al. [106] suggest that studies need to understandably be adapted to the needs and wishes of the participants. These modifications could include simplification of the materials and a shorter duration of the experiments, and lead to difficulties in understanding how responses should be interpreted. Additionally, research has to be carried out in a short period, as a participant’s situation may change rapidly [106]. Their recommendation for future research on the topic is that the main focus should be on understanding the brain in order to gain a better understanding of different systems at a neural level and memory representation of music. In order to obtain the best results, it is suggested to form a closer link between professionals from different disciplines, such as psychology, biomedical sciences, and music therapists.

To help ease the research requirements of proposed solutions and reduce unwanted stress in PwD, the authors suggest to conduct pilot studies with healthy participants in order to validate the technical aspects, before fine-tuning the proposed solution for the needs of PwD. Likewise, it may also be desirable to consider the benefits of listening to music in combination with other enjoyable activities. Samson et al. [6] showed that both cooking and music interventions can improve emotional and behavioural functioning in PwD.

### 4.3. Integrating Sensing Systems and Music

While methodological approaches using music interventions for PwD are not consistent, a trend can be inferred that shows the positive effect music could have on the emotional well-being of PwD. Combining this and emotion-monitoring systems to estimate mental states, an opportunity reveals itself where the mental state of people with dementia can be catered for in a cost-efficient fashion. Such a system that integrates emotion monitoring with music could assess the emotional state of PwD and provide appropriate music. These systems could adapt to personal responses to specific music and optimise automated music interventions when deployed in the long term using reinforced learning methods.

Developing pervasive solutions is often challenging due to the limited amount of power and battery in small devices, which results in challenges such as battery degradation [18,95], general power consumption [35,49], or computational resource constraints, such as limited memory [95]. Unobtrusive solutions require researchers to develop smaller and more efficient technologies that last longer. Hence, Kanjo [49] states that there is a need for efficient algorithms that can extract high-level information from raw data. An additional requirement is that such a system should be easily programmable and highly configurable. This is especially important when taking into account the changing states of people with dementia.

Together with the resource constraints comes the responsiveness (or delay) of such a system. The goal is to minimise the time that is needed to detect a certain change and act on it accordingly [98]. Other challenges regarding pervasive systems include scalability (the ability of systems to handle an increasing amount of users, sensors, or data without compensation in performance) [16] and the limited lifetime of equipment [95].

While networking and distributed computing are outside the scope of this paper, they are integral parts of pervasive computing, as such systems build on the network layer. This is tedious and prone to error. Hence, Taleb [18] stresses that future research avenues should focus on middleware solutions. The aforementioned wireless solutions transfer the collected data using radio waves, meaning humans will be exposed to radio-frequency electromagnetic fields. Although the effects of wireless sensors should be weak [107], their long-term use causes additional concerns. This should be addressed in future research by thinking about the positioning, radiation, and other side effects [16].

It should be noted that the aforementioned ethical considerations (Section 3.4) apply in this context, especially when large amounts of (sensitive) data are communicated between clients and cloud services. Therefore, it is important to ensure user data protection. Another challenge is PwD in later stages, as they might not be aware or comprehend that sensitive data are being collected and analysed. Hence, it is important to clearly report how data protection is achieved for PwD.

The adoption of pervasive systems can also be difficult. As mentioned before, the continuous collection of the data of a subject might cause a “big brother” effect, limiting user acceptance [95]. Moreover, depending on the application, the cost of invalid results may also limit the adoption of such systems. To address the key concepts of privacy put forward by Langheinrich et al. [92] and McNeil et al. [93], it is important PwD are made aware of how a system works and what data are collected. Furthermore, subjects should give their consent for data collection and ideally a system is adaptable to the user’s wishes, i.e., offer modularity to only use a subset of the sensing/data collection capabilities. Users should be in control of their own data and be aware of the flow of data. PwD may still have the capacity to provide consent in earlier stages of dementia, especially if the study to be performed is mildly complex. However, with the progression of the disease, the capacity to give consent may decline and this should be assessed case-by-case while always following the ethical guidelines.

## 5. Conclusions and Recommendations

Global development has lead to the world population growing steadily, with more people living longer. This puts a strain on the existing healthcare system, especially on formal care for older adults. One of the most prominent challenges within older adult care is dementia. Recently, a push has been made to look for non-pharmaceutical solutions to decrease BPSD and improve the quality of life for PwD. Although the effects of dementia differ per individual, music has shown promising and positive effects on the health and well-being of PwD, as well as improving the perceived quality of life of both PwD and formal and informal caregivers.

Pervasive systems have demonstrated their effectiveness in monitoring the mental and physical health of older adults. By integrating sensors with suitable analytical methods and communication channels, it becomes feasible to collect large volumes of data in a continuous and unobtrusive manner. This enables comprehensive tracking of various aspects of health over time.

The focus of this review was to outline the application of musical interventions for PwD, including how to sense indicators of affective states and what analytical techniques to apply to the data in order to estimate emotions. In collecting this information, several challenges were identified and discussed, which are summarised in Table 4. This table implies that many studies should follow a stricter scientific approach, with the need for standardisation and clear guidelines.

When connecting the challenges of emotion monitoring and the effects of music on people with dementia, multiple research recommendations were identified. An important aspect is ensuring the validity of the results (especially with smaller groups of participants) and increasing the generalisability of the proposed solutions. Therefore, future studies should be performed with larger and more diverse groups of participants over longer periods in the field.

While music interventions and pervasive computing have proven their applicability for older adults and PwD, to the best of the authors’ knowledge, there is currently very limited research that combines these elements into one system. By leveraging pervasive computing, emotion-aware music recommendations for PwD could be developed, which would alleviate BPSD. Based on the analysed literature, this direction appears to be a strong opportunity to address the growing costs of dementia and limited availability of caregivers, while not compensating for the quality of care. When designing such a system, it is crucial to consider human-centered design, both for technical and ethical concerns. This will increase the chances of successful adoption of the proposed systems, which is one of the main challenges.

In conclusion, this research has highlighted many opportunities to address the societal problems that dementia is posing. By effectively tackling the challenges identified in this review, society can advance towards the development of innovative systems or interventions. These solutions aim to address the limited availability of care options and alleviate the emotional and financial burdens faced by stakeholders involved in dementia care.

## Figures and Tables

**Table 1:** Overview of the types of sensing that have been used.

Emotion Sensing Method	Reference
On-body sensors	[18,21,22,23,24,25,26,27,28,29,30,31,32,33,34,35,36,37,38,39,40,41,42]
Device free	[22,23,26,31,34,40,42,43,44,45,46,47,48]
Self-report ing	[31,32,33,34,37,38,39,40,42]

**Table 2:** Grouping of the studied papers into machine learning and deep learning.

Method	References
Machine learning	[18,21,22,24,25,26,27,28,29,30,33,34,35,36,37,40,41,42,43,45,48,49]
Deep learning	[22,25,26,30,38,39,41,44,45,46,47]

**Table 3:** Study environment of the papers studied in this work.

Study Environment	References
Lab setting	[18,23,24,26,27,30,34,39]
Office environment	[21,30,46,48]
In home (incl. healthcare homes)	[31,32,40,42,44,47,48]
In car	[33,43]
Outdoor	[29,38]
VR	[28]
Other	[23,25,46]

**Table 4:** Summarised challenges of the main topics in this report.

Emotion detection	Limited generalisability due to the specific application design and limited diversity in current studies.
	Sensor limitations (storage, battery consumption, and occasional loss of connection).
	Computation and responsiveness challenges.
	Scalability.
	Limited research on (side-)effects of radiation.
	A lack of standardisation and clear requirements in research.
	Research is mostly performed in the lab, limited results in natural environments.
	Adoption (privacy considerations and ethical concerns).
	Limited certainty of the findings due to the use of small sample sizes in research.
Music and dementia	A lack of methodological rigour needed for standardisation.
	Limited research on long-term effects.
	Focus on western cultures, limited knowledge of other cultures.
	No heuristics in what is the best intervention, many intervention design choices and trade-offs.
	Adaptability to the limitations of persons with dementia in general and the progressive nature of dementia.
	Strong results obtained by using music, but uncertainty about its added benefit compared to other pleasurable events.
	Research often puts an additional burden on caregivers.

## Data Availability

Not applicable.

## Abbreviations

The following abbreviations are used in this manuscript:

AD	Alzheimer’s disease
ADL	Activities of Daily Living
AI	artificial intelligence
AmI	ambient intelligence
ANOVA	analysis of variance
ANS	autonomic nervous system
BAN	body area network
BP	blood pressure
BPSD	behavioural and psychological symptoms of dementia
BSN	body sensor network
CBF	content-based filtering
CDR	Clinical Dementia Rating
CF	collaborative filtering
CL	contrastive learning
CNN	convolutional neural network
CPU	central processing unit
DNN	deep neural network
ECG	electrocardiography
EDA	electrodermal activity
EDR	electrodermal response
EEG	electroencephalography
EMG	electromyography
GDP	gross domestic product
GSR	galvanic skin response
HR	heart rate
HRV	heart rate variability
IoT	Internet of Things
IR	information retrieval
kNN	k-nearest neighbours
LDA	latent dirichlet allocation
LSTM	long short-term memory
MADM	multiple-attribute decision making
MAP	mean average precision
MEC	mobile edge computing
MLP	multi-layer perceptron
MMSE	Mini-Mental State Examination
MPR	mean percentile rank
MRR	mean reciprocal rank
MRS	music recommender system
NDCG	normalised discounted cumulative gain
NFC	near field communication
NLP	natural language processing
PLSA	probability-based latent semantic analysis
PPG	photoplethysmography
PwD	people with dementia
RMSE	root mean square error
RNN	recurrent neural network
RS	recommender system
RF	random forest
SCL	skin conductance level
SCR	skin conductance response
SMB	smart music box
SNS	sympathetic nervous system
SVD	singular value decomposition
SVM	support vector machine
WHO	World Health Organisation
XIM	eXperience Induction Machine

## Appendix A. Methods of Systematic Literature Review in Detail

### Appendix A.1. Identification of Articles

This state-of-the-art review consists of research into two domains: social sciences and exact sciences. To collect the state-of-the-art literature on the more exact sciences of emotion monitoring, Scopus was chosen. To identify relevant studies on the topic of music and dementia, two electronically available databases in the direction of social sciences were consulted: PsycInfo and Wiley. The search queries are presented in Table A1.

**Table A1:** Search queries used to identify the literature.

Topic	Search Words	Location
The effect of music on PWD	Music	Title
	Dementia	Title
	Emotion	Abstract
Sensing systems for emotional state monitoring	Sensing or sensors	
	Pervasive or unobtrusive or device-free or ubiquitous	N/A

In PsycInfo and Scopus, filters were used to further extend the query search. These filters are shown as search limitations for inclusion and exclusion in Table A2.

**Table A2:** Search limitations for inclusion and exclusion.

PsycInfo	Scopus
Search limitations for inclusion	
Papers that use related words or equivalent subjects	In final publication stage
Peer-reviewed	Peer-reviewed
Publication type is a journal	
Status is fully published	
Search limitations for exclusion	
The paper is not published in English	Paper does not contain one of the keywords in the list

For both topics together, 659 articles were found in total (PsycInfo: 22; Wiley: 12; Scopus: 625).

### Appendix A.2. Selection of Relevant Studies

For the resulting set of literature on emotion monitoring (*n* = 79), filtering was performed in two steps. In the first step (screening), the title and abstracts were surveyed to eliminate irrelevant papers (*n* = 31). Papers were deemed irrelevant if they did not discuss a form of emotion monitoring, or when the topic was too narrowed down (e.g., one specific disorder that is not dementia). This step left 48 papers remaining. In the second step, full-text screening was performed to narrow down the set of papers further. From papers that had overlap between each other (i.e., same author but only partial results), only the most recent ones were included. Additionally, papers that drift too far away from the topic studied in this work or papers that cover small specific elements within the system in too much detail were excluded. This is because of the demarcated time and scope. This step excluded 20 works from the review. After full-text screening, 28 papers were included in this study on the topic of sensing systems for emotional state monitoring.

The resulting set of literature (*n* = 23) from the query search mentioned above on the effect of music on PwD was screened. The criterion to include a paper is that the paper should address the interaction of two topics: music and dementia. Furthermore, three papers were disregarded due to their lack of complete reporting. In the next stage (*n* = 20), full-text screening caused two more papers to be eliminated based their distance from the topic studied in this paper. The set of included work consists of 18 studies for this topic.

## Appendix B. Overview of the Literature on Music Interventions for PwD

The tables to follow in this appendix contain multiple abbreviations. These are listed below.

f	female
m	male
$m_{age}$	mean age
G	group (intervention)
I	individual (intervention)
A	active (music intervention)
P	passive (music intervention)
N/D	no data
N/A	not applicable

**Table A3:** Music interventions for PwD.

Study and Year	N, Age, Gender Availability per Work	Study Setup	Dementia Severity	Active or Passive	Type of Music	Time Span	Results
[6] 2015	2 studies (14, 48 participants), no age or gender given	G	Moderate to severe	A or P	Familiar (French) songs	2 times a week for 4 weeks either 1 or 2 h	Non-pharmacological interventions can improve emotional and behavioural functioning and reduce caregiver distress, but the added benefit of music is questioned.
[12] 2019	1 participant f:1, age: 77	I	Severe	A and P	Familiar music	Retrospective study, musical activities on a daily basis to a few times per week for years	The study suggests that music could be a vital tool in coping with symptoms of severe dementia.
[13] 2009	10 studies 16–60 participants	G and I	Mild to severe	A and P	Hymns, familiar songs, big band, classical, individualised, improvisation	4–30 sessions, or weekly session for 2 years	The review reveals an increase in various positive behaviours and a reduction in negative behaviours, as well as the incorrect use of the term music therapy and lack of methodological rigour.
[14] 2012	30–100 participants	G and I	Mild to severe	A and P	Diverse	Diverse	Short-term studies incorporating music activities have shown positive effects, but a lack of proper methodological foundation and long-term study make these conclusions uncertain and less generalisable.
[67] 2005	6 participants	I	Severe	A	Well-known songs	20 daily sessions of 20–30 min	Results show that individual music therapy is suitable to reduce secondary dementia symptoms and meets the psychosocial needs of patients.
[68] 2013	19 studies 10–55 participants	G and I	Mild to severe	A and P	Varying types of music per paper: classical, popular, selected music, live or recorded, etc.	2 weeks–16, 53 weeks	Music interventions could improve the QoL for PwD, but poor methodological rigour limits confident interpretation of the results.
[63] 2011	100 participants f:53 m:47 $m_{age}$: 81.8	G	Mostly moderate	A and P	rhythmical, slow-tempo, instrumental, personalised, glockenspiel	two 30-min sessions per week for 6 weeks	The experimental group showed better performance (reduction in negative behaviours) after the intervention
[70] 2021	21 studies 9–74 participants $m_{age}$ = 68.9–87.9	G and I	Mild or moderate	A	Diverse	Varying frequencies, durations and langths, from daily to weekly for weeks to months	Music causes a significant effect on the scores of cognitive function of elderly with mild cognitive impairment or dementia and has shown positive effects on mood and quality of life.
[66] 2011	43 participants f: 31, m: 12 $m_{age}$ = 78.2	G	Moderate	A	Tailored, live or recorded	Once a week 6 h intervention for 8 weeks	Results suggest that weekly music therapy and activities can alleviate behavioural and depressive symptoms in PwD.
[75] 2001	9 participants f: 7, m:2 $m_{age}$ = 81	I	Severe	A and P	Familiar, preferred music	6–22 min, 3 sessions p.p. The average observation period for a patient was 13 days but varied between 3 and 49 days.	The use of music (by caregiver or background music) improves communication between the caregiver and patient, causing positive emotions to be amplified, while decreasing aggressiveness.
[77] 2015	9 participants f: 6, m:3 $m_{age}$ = 81	I	Moderate to severe	A and P	Diverse	Weekly sessions of 23–39 min, at least 20 individual sessions over a period of 6 months per participant	Individual music therapy has a significant, positive effect on the communication, well-being, and (positive) emotion expression of people with dementia.
[81] 2016	89 caregivers 84/74 PwD	G and I	Mild to moderate	A and P	Familiar songs	Weekly sessions of 1.5 h for 10 weeks	Both singing and listening can target different depression symptoms.
[82] 2014	16 participants f: 15, m: 1 $m_{age}$ = 87.5	G	Mild to severe	A and P	Patient’s preferences	12 weekly sessions	The intervention does not show a significant change in quality of life, but it does show a significant increase in emotional well-being and a negative significant change in interpersonal relations.
[72] 2012	54 participants f: 30, m:15	G and I	Mild to severe	A and P	Intimate live music, genre preference of audience	45 min, 17 performances divided over a selection of nursing homes	Live music positively affected human contact, care relationships, and positive and negative emotions.
[59] 2009	23 participants f: 15, m: 8 $m_{age}$ =73	I	Mild	P	Novel instrumental clips from the film genre	1 time session with 3 tasks	Alzheimer patients show well-preserved emotional recognition of music.
[61] 2015	26 participants f:6, m: 20 $m_{age}$ = 64	I	Frontotemporal dementia	P	Based on four-note arpeggio chords and wave files of human nonverbal emotional vocalisations	1 session with multiple trials per condition	The research suggests that music can delineate neural mechanisms of altered emotion processing in people with dementia.
[108] 2012	N/D	G and I	N/D	A and P	N/D	N/A	The literature reflection shows that music has a positive effect on people with dementia, but also questions the flexibility of methods with respect to changes in participants with dementia.
[106] 2012	N/D	G	N/D	N/D	N/D	N/D	The article points to multiple studies that support the power of music, but also unveils challenges in research with dementia patents such as recruitment, narrow time windows, burden on participants and carers, and tailored research.

## Appendix C. Overview of Literature on Emotion Sensing

The tables to follow in this appendix contain multiple abbreviations. These are listed below.

f	female
m	male
$m_{age}$	mean age
fr	frequency
d	duration
AI	Artificial Intelligence
AIC	Akaike Information Criterion
ANN	Artificial Neural Network
ANOVA	Analysis of Variance
ARM	Association Rule Mining
CMIM	Conditional Mutual Information Maximisation
CNN	Convolutional Neural Network
CSI	Channel State Information
cvx-EDA	Convex (optimisation) Electrodermal Activity
DISR	Domain-Invariant Subspace Representation
DNN	Deep Neural Network
DT	Decision Tree
ECG	Electrocardiogram
EDA	Electrodermal Activity
EDR	Electrodermal Response
EEG	Electroencephalogram
ELM	Extreme Learning Machine
EMG	Electromyography
FMCW	Frequency-Modulated Continuous Wave
FMF	Fuzzy Membership Functions
fMRI	Functional Magnetic Resonance Imaging
fNIRS	Functional Near-Infrared Spectroscopy
GDA	Gaussian Discriminant Analysis
GP	Genetic Programming
GPS	Global Positioning System
GBC	Gradient Boosting Classifier
GLM	Generalised Linear Model
GSR	Galvanic Skin Response
HMD	Head-Mounted Display
HR(V)	Heart rate (variability)
IMU	Inertial Measurement Unit
JMI	Joint Mutual Information
KBCS	Kernel-based Class Separability
kNN	k-Nearest Neighbours
LDA	Linear Discriminant Analysis
LDC	Linear Discriminant Classifier
LMM	Linear Mixed Models
LR	Logistic or Linear Regression
LSTM	Long Short-Term Memory
MANOVA	Multivariate Analysis of Variance
ML	Machine Learning
MLP	Multi-Layer Perceptron
NB	Naive Bayes
NN	Neural Network
PCA	Principal Component Analysis
PNN	Probalistic Neural Network
PPG	Photoplethysmogram
PSD	Position sensitive device
PTT	Partial Thromboplastin Time
RBF	Radial Basis Functions
RF	Random Forest
RFE	Recursive Feature Elimination
RNN	Recurrent Neural Network
RR	Respiration Rate
RSS	Received Signal Strength
RVM	Relevance Vector Machine
QDA	Quadratic Discriminant Analysis
SD	Standard Deviation
SDK	Software Development Kit
SFFS	Sequential Forward Floating Selection
(L-)SVM	(Linear) Support Vector Machines
WiFi	Wireless Fidelity

**Table A4:** Emotion monitoring systems—Experiments.

Study and Year	Aim	Features	Measuring	Sensors Used	Study Environment	*n*	Algorithm(s)	Results
[18] 2010	Propose a framework to realise ideas and describe their effective functions in drug addiction treatment application scenarios for roaming patients.	Sensor proxy, pulse, blood pressure, breathing, body temperature	Response to drugs/drug overdose avoidance	Laptop-like wearable and biomedical sensors	Test-bed setting, simulations	1 patient, 20 responders	Data aggregation and decision: user profile, local resuer selection: multi-attribute decision making algorithm	A (drug overdose) case study showed promising results in monitoring psychophysiological conditions, aggregating this data and prompting assistance. PEACH is viable in terms of its responsiveness, battery consumption, and memory use.
[21] 2020	Explore whether perception clustering can be used to reduce the computational requirements necessary for personalised approaches to automated mood recognition.	Heart rate, pulse rate, pulse-wave transit time, skin temperature, and movements	Excited, happy, calm, tired, bored, sad, stressed, angry	ECG, PPG, and accelerometer	Work	*n* = 9	k-means/hierarchical clustering, RFE with LR, classifiers (BE-DT, DT, kNN, LR, L-SVM, SVM-RBF, regression (GP)	Perception clustering is a compromising approach that reduces computational costs of personal models, while performing equally better than generalised models.
[24] 2021	Investigate the effect of emotional autobiographic memory recall on a user’s physiological state in VR using EDA and pupil trackers.	Pupil physiological signals and sweat conductance features	Emotional autobiographic memory recall (positive, negative and neutral) in VR	EDA (Shimmer3 GSR+ sensor) + Eye tracker (Vive Eye tracking SDK, Vive Pro Eye HMD)	Lab	*n* = 6 (f: 2, m: 4, $m_{age}$ = 26.8)	Statistics (Friedman tests, Nemenyi post hoc tests, Wilcoxon-Signed Ranks tests, Shapiro–Wilk test)	EDA and pupil diameter can be used to distinguish autobiographic memory recall from non-AM recall, but no effect was found on emotional AM recall (positive, negative, neutral).
[27] 2021	Validate a self-developed algorithm that recognises emotions using a glasses-type wearable with EDA and PPG.	Facial images and biosignals (heart rate features and skin conductance)	Arousal and valence, or categorical: amusement, disgust, depressed, and calm	Glasses-type wearable with EDA, camera and PPG	Shield room	*n* = 24, ($m_{age}$ = 26.7)	PCA, LDA, and SVM with RBF kernel or binary RBF SVM model	The glass-type wearable can be used to accurately estimate the wearer’s emotional state.
[28] 2021	Present a VR headmount with a multimodal approach to provide information about a person’s emotional and affective state, addressing low latency detection in valence.	Facial muscle activation, pulse measurements, and information on the head and upper body motions	Affective state and context (i.e., HRV, arousal, valence, and expressions)	f-EMG, PPG, and IMU	VR	Not mentioned (demo)	AI and ML	Not available (yet)
[29] 2021	Reduce computational complexity while increasing performance in estimating tourist’s emotions and satisfaction.	Eye, head, and body movements, eye gaze, facial and vocal expressions, weather information	Tourist emotion and satisfaction.	Smartphone (camera) and Pupil Labs Eye Tracker, SenStick multi-sensor board	Sightseeing in Ulm in Germany, Nara and Kyoto in Japan	*n* = 22 + 24	PCA, SVM, RBF	The newly suggested model outperforms a previous method by the authors. Additionally, the inclusion of weather conditions improves model accuracy.
[30] 2021	Design a framework consisting of stress detection and emotion recognition to maintain productivity and workability while balancing work and private life in ageing workers unobtrusively.	Stress: statistical properties of biosignals (ECG and HRV signals) and normal-to-normal differences or 2D ECG spectrograms. Emotions: Facial images	Stress and emotion (Positive, negative, and neutral)	ECG, EDA, mobile phone camera	Office, lab setting	Stress: 25 and 15 subjects. Emotion: 28,000 images.	Stress: CNN and different classifiers (SVM, kNN, RBF, RF, ANN). Emotion: CNN	The presented framework outperforms previous work on the recognition of stressful conditions, mainly in the office environment, using EDA and ECG biosignals or facial expression patterns.
[31] 2018	Validation and experiments of ORCATECH [40] and determining what is feasible.	Bed(room) activity, sleep (d), computer use (fr+d), motor speed and typing speed, medication taking behaviour, walking velocity, visitors (fr.+d), social activities on internet (d), out of the home (d), phone calls (fr), driving, body composition, pulse, temperature, and $CO_{2}$	Sleep, computer use, medication adherence, movement patterns and walking, social engagement	IR sensor, magnetic contact sensors, pc, optional: medication trackers, phone monitors, wireless scales, pulse, temperature and air quality and undisclosed driving sensors	In real homes	*n* = 480 homes	Update on [40] surveying multiple algorithms	An update on [40]: ORCATECH provides continuous unobtrusive monitoring that seems to be more widely supported in elderly homes
[32] 2018	Investigate the use of smartphones/smartwatches to measure continuously and objectively and provide personalised support in rehabilitation.	Location, activity, step count, battery status, screen on/off	Behaviour analysis for rehabilitation	Smartphones and smartwatches	Real-life setting, participants required to live with caregiver	*n* = 6 PwD (m: 4, f: 2), ages: 68–78	Location: density-based clustering, activity duration: filtering and grouping	The authors claim this is the first evidence describing the role of personal devices in dementia rehabilitation. Data gathered in real life are adequate for revealing behaviour patterns. Potential advantages of sensor-based over self-reported behaviour
[33] 2020	Reflect on two studies: (1) link between cardiovascular markers of inflammation and anger while commuting and (2) investigate the effect on the heart rate.	IBI, PI, driving features, HR(V), PTT	Anger monitoring in commuter driving	Smartphone, Shimmer3 accelerometer, two Shimmer3 sensors (ECG and PPG)	Actual driving	(1) 14 (f: 7), age 25–57, (2): 8 (f: 6), age 28–57	Independent evaluation of driving and physiology features, then ensemble classifier utilising LDA, Decision Tree, kNN classifiers. Study 2 is statistical analysis.	Accuracy of >70% in anger detection using physiology and driving features. The means of HR and power in high frequencies of HRV and PTT were sensitive to subjective experience of anger.
[34] 2014	Proposing a hybrid approach to create a space with the advantages of a laboratory within a real-life setting (eXperience Induction Machine—XIM).	HR(V), skin conductance, acceleration, body orientation	Arousal and response to environment	Multi-modal tracking system, pressure sensors, microphones, sensing glove and shirt (EDR, ECG, respiration, gestures)	eXperience Induction Machine (real-life setting replicated with laboratory advantages)	Study 1: 7 (f: 4, m: 3), age: 29.7 ± 3.9 (SD), Study 2: 11 (f: 7, m: 4), age: 27 ± 4.51 (SD)	Linear Discriminant Classifier (LDC)	The authors claim it is possible to induce human-event-elicited emotional states through measuring psychophysiological signals under ecological conditions in their space
[35] 2015	Propose and evaluate textile low-powered biofeedback wearables for emotional management to transform negative emotional states into positive.	ECG and respiratory rate, for HRV and RR (patterns)	Negative emotions	Self-designed textile wearable with ECG and respiration sensor	University	*n* = 15, ages 20–28,	Statistical analysis (ANOVA)	The authors developed a low-powered, low-complexity textile biofeedback system that measures real-time HRV, wirelessly connects to laptops/phones, and is statistically effective in cases of negative emotion.
[36] 2019	Validation and experimental results (continuation of previous research) of a wearable ring to monitor the ANS through EDA, heart rate, skin temperature, and locomotion	Electrodermal activity/galvanic skin resistance, heart rate, motor, temperature	Stress levels are measured as an experiment in students	Ring with HR sensor and pulse oximeter, accelerometer, temperature sensor, skin conductance electrodes, wireless microprocessor	-	*n* = 43 (f: 20, m: 23), ages 19–26	Decision tree, support vector machine, extreme gradient boosting	Stress levels monitored with 83.5% accuracy using SVM (not mentioned how many classes/different stress levels)
[37] 2017	Introduction of wearable device for calm/distress conditions using EDA with classification accuracies and validation	Electrodermal activity (EDA) (temporal, morphological, frequency)	Calm or distress	10 mm silver electrodes on the fingers (palm sides index and middle finger)	Not mentioned, but likely a lab given the prototype	*n* = 45 (f: 20, m: 25), age 23.54 + 2.64 (based on 50–5 were invalid)	Statistical analysis (ANOVA), decision trees	89% accuracy for differentiating between calm or distress for combined features, with individual features this was (much) lower.
[38] 2018	Estimation and evaluation of emotional status/satisfaction of tourists by using unconscious and natural actions.	Location, vocal and facial expressions, eye gaze, pupil features, head tilt, and body movement (footsteps)	Positive: excited, pleased, calm; neutral: neutral; negative: tired, bored, disappointed, distressed, alarmed	Smartphone for video and audio, eye-tracking headset, sensor board SenStick with accelerometer, gyroscope, GPS	Real-world experiments	*n* = 22 (f: 17, m: 5), ages 22–31, $m_{age}$ = 24.3	RNN-LSTM with RMSProp optimiser	Unweighted Average Recall (UAR) of 0.484 for Emotion, and Mean Absolute Error (MAE) of 1.110 for Satisfaction based on the three emotion groups (rather than all nine emotions)
[39] 2017	Propose an emotion recognition system for affective state mining (arousal) using EMG, EDA, ECG.	Arousal and valence (skin conductance level, frequency bands of parasympathetic and sympathetic signals and impulses of the zygomaticus muscle)	Happy, relaxed, disgust, sad, neutral	Biomedical sensors (EDA, ECG, facial EMG)	DEAP [109]	DEAP (*n* = 32 total)	Fusion of the data and then a convolutional neural network	87.5% using the proposed CNN which at the time was the best state-of-the-art method
[41] 2013	Develop a novel, wearable mood recognition system using ANS biosignals for mood recognition in bipolar patients	Inter-beat interval series, heart rate, and respiratory dynamics, ECG, RSP, body activity	Remission euthymia (ES), mild depression (MD), severe depression (SD), mild mixed state (MS)	PSYCHE platform sensorised t-shirt, textile ECG electrodes, piezoresistive sensor, accelerometer	Followed for 90 days, real-life scenario	*n* = 3, age = 37–55 with bipolar disorder (I or II)	PCA. linear and quadratic discriminant classifier, mixture of Gaussian, kNN, Kohonen self organising map, MLP, probabilistic neural network	Good classification accuracy (diagonal) in the matrices. It is stated that it is better at distinguishing between euthymia and severe states compared to mild states due to their closer resemblance
[42] 2009	Exploration of the relationship between physiological and psychological variables measured at home manually or automatically	Activity, weight, steps, bed occupancy, HR, RR, blood pressure, room illumination, temperature; (self-assessment) stress, mood, exercise, sleep	Stress levels	Activity monitor, HR monitor, mobile phone, step counter, blood pressure monitor, personal weight scale, movement sensor in bed, temperature and light sensors	Rehabilitation centre	*n* = 17 (f: 14, m: 3), age 54.5 ± 5.4 (SD)	Statistical analysis (Spearman correlations)	Unobtrusive sensors measure significant variables, but overall modest correlation with self-assessed stress level when combined with other participants. Overall, there are strong correlations, but these may conflict on a personal level.
[43] 2021	Evaluate VEmotion, aiming to predict driver emotions using contextual data in an unobtrusive fashion in the wild.	Facial expression, speech, vehicle speed and acceleration, weather conditions, traffic flow, road specifications, time, age, initial emotion	Driver emotions (neutral, happy, surprise, angry, disgust)	Smartphone: GPS, camera, microphone	In-car	*n* = 12 (m: 8, f: 2, $m_{age}=27$)	Random Forest Ensemble Learning based on 10-fold grid-search cross validation (using SVM, kNN, DT, Adaboost, and RF from scikit-learn with default parameters)	Context variables can be captured in real time using GPS at low cost, optionally accompanied by camera monitoring the driver “in-the-wild”, specifically in-vehicle.
[44] 2021	Measure audience feedback/engagement of entertaining content with using ultrasound and echoes of the face and hand gestures.	(Echoes of) facial expressions	User engagement: six basic emotions combined with hand gestures	SonicFace (Speakers + microphones) ->ultrasound	Home environment (in front of screen)	*n* = 12 (f: 2, m: 10, $m_{age}=26$)	FMCW-based absolute tracking, beam-forming, multi-view CNN ensemble classifier	SonicFace reaches an accuracy of almost 80% for six expressions with four hand gestures and has shown robustness and generalisability in evaluations with different configurations.
[45] 2016	The authors propose a method to use facial expression and gestures to detect emotions using video data and self-implemented software approaches.	Facial expressions according to CANDIDE face model	Happiness, relaxed, sadness, anger	Microsoft Kinect (Emotion Recognition Module)	Likely lab, but undisclosed	*n* = 2	Genetic programming, voting, and multiple classifiers selected	Average accuracy of around 68% for test set (out of normal voting/evolved voting and combined/uncombined valance/arousal evolving subtrees)
[46] 2021	Propose a domain-independent generative adversarial network for WiFi CSI-based activity recognition.	Raw CSI	Clap, sweep, push/pull, slide	WiFi-CSI	Classroom, hall and office	*n* = 17 (widar3.0 database))	Adversarial domain adaptation network (CNN-based feature extraction and simplified CSI data pre-processing)	The ADA pipeline showed superior results compared to current models in activity recognition using WiFi-CSI in terms of robustness against domain change, higher accuracy of activity recognition, and reduced model complexity.
[47] 2020	Establishing a mental health database; proposing a multimodel psychological computational technology in universal environment	Facial expression (gaze), speech (emotion detection)	Short-Term Basic emotions, long-term complex emotions and suspected mental disorders (uni- and bipolar DD, schizophrenia)	Camera, microphone	Mental health centre	*n* = 2600 (f: 405) over 12 scenarios	Multimodal deep learning for feature extraction, then input into LSTM with attention mechanism	State-of-the-art performance in emotion recognition, identified continuous symptoms of three mental disorders, now quantitatively described by a newly introduced model; established relationship between complex and basic emotions.
[48] 2020	Exploring new combinations of parameters to assess people’s emotion with ubiquitous computing	Speech (variability of frequency of pitch, intensity, energy) and ECG (heart rate variability)	Arousal–valence model, the six basic emotions	ECG belt, multifunction headset with microphone	50% in office environment, 50% in living room environment	*n* = 40 (f: 20, m: 20), age: 27.8 ± 7.6 (SD), range 18–49	Statistical analysis with MANOVA (Wilks’ lambda) and ANOVA (Huynh-Feldt)	The aforementioned speech parameters combined with HRV provide a robust, reliable, and unobtrusive method of reflecting on a user’s affective state.

**Table A5:** Emotion monitoring systems—Surveys.

Study and Year	Aim	Features	Type of Emotions Recognised	Sensors Used	Study Environment	*n*	Algorithm(s) Used	Results
[22] 2021	Present an elaborate overview of approaches used in emotion recognition and discussions of ethics and biases.	Facial expressions, speech (pitch, jitter, energy, rate, length, number of pauses), activity, heart rate (variability), galvanic skin response, eye gaze and dwell time, user’s location, time, weather and temperature, social media	Stress, depressive symptoms, user experience/user engagement, mental health, anger, anxiety, and more	Camera, IMU, EDA, ECG, PPG, EEG, microphone, fNIRS, accelerometer, gyroscope, magnetometer, compass	N/A	N/A	Regression analysis, SVM, predictive and descriptive models, DT, clustering, kNN, naive Bayes, RF, NN	A design space for emotion recognition is created and based on the literature it was found that the approach is decided based on domain-specific user requirements, drawbacks, and benefits.
[23] 2022	Review state-of-the-art unobtrusive sensing of humans’ physical and emotional parameters comprehensively.	Natural signals (e.g., heat, breathing, sound, speech, body image (facial expression, posture)) and artificial signals (i.e., signal reflection and signal interference by the body).	Activity, vital signs, and emotional states	Geophone, finger oximeter, camera, smartphones, earphone, microphone, PPG, infrared, thermography, (FMCW) radio wave antennas, RSS, PSD, CSI	Diverse (lab, hospital, ambulance)	N/A	N/D	The paper provides a taxonomy for human sensing. Remaining challenges of human sensing include amongst others noise reduction, multi-person monitoring, emotional state detection, privacy, multimodality and data fusion, standardisation, and open datasets
[25] 2022	Review IEEE research articles from the last 5 years that study affective computing using ECG and/or EDA thorougly.	Heart rate and skin conductance features	Stress, fear, valence, arousal, emotions	ECG and EDA	Diverse	*n* = 27 papers, 18–61 people per study included in this paper	cvxEDA, DT, PCA, kNN, (Gaussian) Naive Bayes, (L)SVM (with RBF), LDA, (M)ANOVA, LMM, SFFS-KBCS-based algorithm, GDA and LS-SVM, R-Square and AIC, RVM, ANN, CNN, RNN, JMI, CMIM, DISR, ELM	The authors argue that EDA and ECG will become a vital part of affective computing research and human lives, since the data can be collected comfortably using wearables and a(n upcoming) decrease in costs, but the current literature on the topic is limited compared to EEG.
[26] 2022	Provide a comprehensive review of signal processing techniques for depression and bipolar disorder detection.	Heart activity, brain activity, typing content, phone use (e.g., typing metrics, number of communications, communication timings and length, screen on/off or lock data), speech, movement, location, posture, eye movement, social engagement	Depressive moods and manic behaviours	Clinical sensors (fNIRS, fMRI), ubiquitous sensors (electrodes (EEG/ECG), software, accelerometer, microphone, camera, GPS, WiFi)	Clinical setting	Depression: 13–5319 people per study included in this work. Bipolar: 2–221	SVM, CNN, RF, Logistic and linear regression, Gradient Boosting Classifier, kNN, RNN, LSTM, MLP, Fuzzy Membership Functions, Adaboost, GLM, Naive Bayese, ANN, ARM, DNN, Gradient Boosting Classifier, Semi-Supervised Fuzzy C-Means, QDA	Two areas of improvement remain: dataset imbalance and the need to move towards regression analysis. Four challenges in disorder detection: clinical implementation, privacy concerns, lack of study towards bipolar disorder, and lack of long term longitudinal studies.
